# Mitigating the economic burden of GnRH agonist therapy for progestogen-resistant endometriosis: why not?

**DOI:** 10.1093/hropen/hoad008

**Published:** 2023-03-14

**Authors:** Paolo Vercellini, Veronica Bandini, Laura Buggio, Giussy Barbara, Nicola Berlanda, Dhouha Dridi, Maria Pina Frattaruolo, Edgardo Somigliana

**Affiliations:** Department of Clinical Sciences and Community Health, Università degli Studi, Milano, Italy; Department of Obstetrics and Gynecology, Fondazione IRCCS Ca’ Granda Ospedale Maggiore Policlinico, Milano, Italy; Department of Clinical Sciences and Community Health, Università degli Studi, Milano, Italy; Department of Obstetrics and Gynecology, Fondazione IRCCS Ca’ Granda Ospedale Maggiore Policlinico, Milano, Italy; Department of Clinical Sciences and Community Health, Università degli Studi, Milano, Italy; Department of Obstetrics and Gynecology, Fondazione IRCCS Ca’ Granda Ospedale Maggiore Policlinico, Milano, Italy; Department of Obstetrics and Gynecology, Fondazione IRCCS Ca’ Granda Ospedale Maggiore Policlinico, Milano, Italy; Department of Obstetrics and Gynecology, Fondazione IRCCS Ca’ Granda Ospedale Maggiore Policlinico, Milano, Italy; Department of Obstetrics and Gynecology, Fondazione IRCCS Ca’ Granda Ospedale Maggiore Policlinico, Milano, Italy; Department of Clinical Sciences and Community Health, Università degli Studi, Milano, Italy; Department of Obstetrics and Gynecology, Fondazione IRCCS Ca’ Granda Ospedale Maggiore Policlinico, Milano, Italy

**Keywords:** endometriosis, pelvic pain, GnRH agonists, triptorelin, leuprorelin, systematic review

## Abstract

**STUDY QUESTION:**

Is it possible to reduce the cost of GnRH agonist treatment for endometriosis by using non-standard dosing regimens?

**SUMMARY ANSWER:**

An extended-interval dosing regimen of a 3.75 mg depot formulation of triptorelin injected every 6 weeks instead of every 4 weeks reduces the cost by one-third without compromising the effect on pain relief.

**WHAT IS KNOWN ALREADY:**

Cost constitutes a limit to prolonged GnRH agonists use. Alternative modalities to reduce the economic burden of GnRH agonist treatment have been anecdotally attempted.

**STUDY DESIGN, SIZE, DURATION:**

A systematic review was conducted to evaluate and compare the effect of three alternative modalities for GnRH use in women with endometriosis, i.e. intermittent oestrogen deprivation therapy, reduced drug dosage, and extended-interval dosing regimens of depot formulations. A PubMed and Embase search was initially conducted in October 2022 and updated in January 2023 using the following search strings: (endometriosis OR adenomyosis) AND (GnRH-agonists OR gonadotropin-releasing hormone agonists OR triptorelin OR leuprorelin OR goserelin OR buserelin OR nafarelin). Full-length articles published in English in peer-reviewed journals since 1 January 1980, and reporting original data on GnRH agonist treatment of pain symptoms associated with endometriosis were selected.

**PARTICIPANTS/MATERIALS, SETTING, METHODS:**

Information was extracted on study design, GnRH-agonist used, dosage, total duration of therapy, side effects, treatment adherence, and pelvic pain relief. Reviews, commentaries, conference proceedings, case reports, and letters to the editor were excluded.

**MAIN RESULTS AND THE ROLE OF CHANCE:**

Of the 1664 records screened, 14 studies regarding clinical outcomes associated with the 3 considered alternative modalities for GnRH agonist use were eventually included (intermittent oestrogen deprivation therapy, n = 2; low-dose or ‘draw-back’ therapy, n = 8; extended-interval dosing regimen, n = 4). Six studies were randomized controlled trials (RCTs) (double blind, n = 2) and eight adopted a prospective cohort design (non-comparative, n = 6; comparative, n = 2). A total of 776 women with endometriosis were recruited in the above studies (intermittent oestrogen deprivation therapy, n = 77; low-dose or ‘draw-back’ therapy, n = 528; extended-interval dosing regimen, n = 171). Robust data demonstrating cost saving without detrimental clinical consequences were available for the extended-interval dosing regimen only. In particular, the 3.75 mg triptorelin depot preparation inhibits ovarian function for a longer period compared with the 3.75 mg leuprorelin depot preparation, allowing injections every 6 instead of 4 weeks. Based on the cost indicated by the Italian Medicine Agency for the 3.75 mg triptorelin depot preparation, this would translate in a yearly saving of €744.60 (€2230.15–€1485.55; −33.4%).

**LIMITATIONS, REASONS FOR CAUTION:**

The quality of the evidence reported in the selected articles was not formally evaluated and a quantitative synthesis could not be performed. Some studies were old and the tested therapeutic approaches were apparently obsolete. Only cost containment associated with GnRH analogue use, and not cost-effectiveness, has been addressed.

**WIDER IMPLICATIONS OF THE FINDINGS:**

Consuming less resources without negatively impacting on health outcomes carries ethical and practical implications for individuals and the community, as this approach may result in overall increased healthcare access.

**STUDY FUNDING/COMPETING INTEREST(S):**

This study was supported by the Italian Ministry of Health (Ricerca Corrente 2023, IRCCS Ca' Granda Ospedale Maggiore Policlinico Milano). E.S. discloses payments from Ferring for research grants and honoraria from Merck-Serono for lectures. All other authors declare they have no conflict of interest.

**REGISTRATION NUMBER:**

N/A.

WHAT DOES THIS MEAN FOR PATIENTS? Some patients with pain symptoms associated with endometriosis do not respond to so-called first-line medications, that is, progestogens with or without oestrogens. In these cases, an alternative class of therapeutics, called GnRH agonists, may be used instead of resorting to surgery. These drugs inhibit ovarian oestrogen synthesis and are generally effective in relieving pelvic pain. When combined with very-low-dose oestrogen–progestogen combinations, to prevent vasomotor symptoms and bone resorption, GnRH agonists may be used indefinitely. Nonetheless, the high cost is a limit to such prolonged treatments.

GnRH agonists are currently delivered via intramuscular depot preparations to be injected every 4 weeks. However, the effect of a specific depot preparation, i.e. the 3.75 mg triptorelin sustained-release formulation, persists for at least 7 weeks and, hence, can be safely injected every 6 instead of 4 weeks, thus allowing for saving of one-third of the expenditure without compromising efficacy.

## Introduction

Endometriosis is commonly associated with pain symptoms severe enough to interfere with daily activity and deteriorate health-related quality of life (HR-QoL). Surgery and hormonal therapy are the treatment options available for endometriosis-associated pelvic pain. Conservative surgical treatment is generally effective, but the magnitude of the benefit is variable and often not long-lasting (Guo, 2009; Vercellini *et al.*, 2009a). Therefore, medical therapy is frequently needed as an alternative to surgery or as a post-operative intervention to prevent lesion and symptom recurrence.

Women not immediately seeking conception and without absolute surgical indications (e.g. subocclusive bowel endometriosis, obstructive uropathy, adnexal mass of doubtful nature, large endometriomas) may use several hormonal medications to inhibit ovulation, interrupt menstruations, and induce a stable endocrine environment. However, hormonal drugs are not cytoreductive and may control, but not definitively cure, endometriosis. Therefore, pain symptoms are generally relieved during treatment, but commonly recur at drug discontinuation. Accordingly, until a curative drug is developed, available suppressive medical therapies for this chronic inflammatory disorder should be used for prolonged periods of time, or until a pregnancy is desired. It follows that the importance of adequately integrating not only efficacy, safety, and tolerability, but also cost, cannot be overemphasized.

Current authoritative guidelines recommend the use of progestogens with or without oestrogen as first-line medications, as they are generally safe, effective, sufficiently tolerated, and inexpensive (National Guideline Alliance (UK), 2017; Becker *et al.*, 2022). However, between one-quarter and one-third of patients do not respond to or tolerate these agents. In these circumstances, GnRH agonists and antagonists are recommended as second-line medications. (National Guideline Alliance (UK), 2017; Becker *et al.*, 2022).

Both classes of drugs induce hypo-oestrogenic states of varying degrees that, on one side, induce substantial temporary lesion inactivity and regression with optimal effects on pain symptoms, but on the other side, also cause adverse effects typical of pharmacological pseudo-menopause such as, among others, vasomotor symptoms, vaginal dryness, psychological complaints, reduced libido, and a decline in bone mineral density (BMD). Thus, GnRH agonists and antagonists should be combined with so-called add-back therapies, such as tibolone or low-dose oestrogen–progestogen combinations that are used for hormone replacement during the menopausal period. The adjunct of add-back therapies to GnRH analogues allows safe and generally well-tolerated extension of treatment courses without the obvious detrimental effects on pain relief.

However, cost remains a major disadvantage of these second-line medications. In Italy, the cost of a 1-year treatment with a depot triptorelin acetate (triptorelin) or leuprolide acetate (leuprorelin) formulation or relugolix (the only GnRH antagonist currently licenced by the European Medicine Agency), combined with add-back therapy, is well above €2000 (£1759; $2126).

In the past, much research has focused on the efficacy, safety, and tolerability of hormonal drugs used to treat endometriosis, but very little attention has been paid to the costs to be borne by patients and their families or by public health services. This sort of nonchalance towards financial implications has probably been favoured by the classic 6-month course paradigm adopted in most trials conducted to test different medical treatments. However, few-month treatments may become some-year treatments when drugs developed within randomized controlled trials (RCTs) are subsequently marketed and implemented in real-world clinical practice. Indeed, studies are already being conducted to assess the impact of relugolix combined with add-back therapy used for 80 instead of the classical 24 weeks in women with endometriosis (Giudice *et al.*, 2022).

Access to healthcare may be linked to socioeconomic status (Fourquet *et al.*, 2019). In low-resource settings, some women may even forgo medical therapy for endometriosis because of excessive costs (Vercellini *et al.*, 2018a), and it is uncertain whether less wealthy public health services in lower-income countries may afford providing prolonged treatments with GnRH analogues (Fourquet *et al.*, 2019). Ideally, the medical approach to endometriosis should be inclusive, economically sustainable, and not elitist. Unfortunately, this may not be the case with GnRH agonists. If access to costly medication for endometriosis is influenced by socioeconomic factors, differences in appropriate care may give cause to health inequalities (Fourquet *et al.*, 2019).

Given that resorting to GnRH agonists is often necessary, containing costs for this class of drugs may allow more women to benefit from appropriate treatment. Moreover, even in settings where public healthcare services provide free GnRH agonists for a limited number of months, reducing the cost of therapy could allow prolonging the period of reimbursed treatment. In general, consuming less resources without negatively impacting on health outcomes carries ethical, as well as practical, implications for individuals and the community, as this approach may result in increased healthcare access for citizens as a whole.

Indeed, in the past years, attempts have been made to elaborate GnRH agonist treatment schedules potentially allowing substantial savings without impairment of pain relief. Specifically, three alternative modalities for GnRH use have been evaluated, i.e. (i) intermittent oestrogen deprivation therapy, (ii) reduced drug dosage, and (iii) extended-interval dosing regimens of sustained-release depot formulations.

Given this scenario, we decided to undertake a systematic review of the available evidence published in the last decades on alternative modalities of GnRH agonists use for symptomatic endometriosis, to verify whether it would be possible to reduce pharmacological expenditure without compromising efficacy and, if this is the case, which is the best therapeutic approach in terms of trade-offs between financial, disease and treatment burdens. Alternative modalities for GnRH antagonist use, if any way possible, are not considered here, as published evidence on this class of drugs is too recent and limited.

## Materials and methods

The objective of the present review was to identify studies aimed at reducing the cost of GnRH agonist treatment in women with symptomatic endometriosis and to assess whether the adoption of intermittent oestrogen deprivation therapy (i.e. a classic 6-month course of a GnRH agonist followed by further 3-monthly courses of the same regimen only in case of recurrence of moderate to severe pain symptoms), low-dose or ‘draw-back’ therapy (i.e. initial achievement of complete pituitary desensitization using a standard GnRH agonist dose for a brief treatment period, and subsequent long-term tapering of the drug dose), or extended-interval dosing regimen (i.e. extending the interval of GnRH agonist sustained-release depot formulations administration) modalities is associated with similar pain relief compared with standard treatment modalities. An overview of the available literature was undertaken following the Preferred Reporting Items for Systematic Reviews and Meta-Analyses (PRISMA; Page *et al.*, 2021). As published de-identified data were used, this study was exempt from ethical approval.

A primary MEDLINE search through PubMed and Embase was initially conducted in October 2022 and updated in January 2023 using the following search strings: (endometriosis OR adenomyosis) AND (GnRH-agonists OR gonadotropin-releasing hormone agonists OR triptorelin OR leuprorelin OR goserelin OR buserelin OR nafarelin). Full-length articles published in English in peer-reviewed journals since 1 January 1980, were selected. Only studies conducted with human participants were considered. In the case of multiple articles originating from the same study, only the one with the most recent data or with more detailed information was included. Reviews, commentaries, conference proceedings, case reports, and letters to the editor were excluded. Discordances between the two investigators were resolved by discussion.

Two authors (P.V. and V.B.) independently assessed the titles and abstracts and selected the articles to be evaluated. References of the retrieved articles were systematically scrutinized to identify additional relevant studies. Further articles were searched using the ‘similar articles’ function in PubMed. Only studies reporting original data on GnRH agonist treatment of pain symptoms associated with endometriosis were selected. Information was extracted on study design, number of treated patients, type of GnRH-agonist used, dosage and number of daily administrations, total length of therapy, side effects, treatment adherence, and degree of relief of pelvic pain symptoms.

Owing to the extreme heterogeneity of treatment schedules adopted, clinical outcomes considered, modalities used to measure pain symptoms and HR-QoL, and length of follow-up planned, a narrative instead of a quantitative synthesis approach was adopted for the present review. The quality of the information reported in the selected reports was not formally assessed.

Additional articles addressing the biological rationale underpinning each individual alternative GnRH agonist use were also reviewed and summarized, although a systematic search was not conducted for this category of publications, as some deal with pharmacokinetic and pharmacodynamic aspects only, and clinical outcomes specifically in women with endometriosis were not measured. A formal protocol was not prepared for this review.

## Results

Of the 1664 records screened, 14 studies regarding clinical outcomes associated with the three considered alternative modalities for GnRH agonist use were eventually included: intermittent oestrogen deprivation therapy, n = 2 (Adamson *et al.*, 1997; Hornstein *et al*, 1997); low-dose or ‘draw-back’ therapy, n = 8 (Hull and Barbieri, 1994; Jacobson *et al.*, 1994; Bergqvist *et al.*, 1997; Uemura *et al.*, 1999; Tahara *et al.*, 2000; Akira *et al.*, 2009; Tang *et al.*, 2017; Harada *et al*, 2022); and extended-interval dosing regimen, n = 4 (Tse *et al.*, 2000; Wong and Tang, 2004; Kang *et al.*, 2010; Liu *et al.*, 2016). Six studies were RCTs (double blind, n = 2), whereas eight adopted a prospective cohort design (non-comparative, n = 6; comparative, n = 2). A total of 776 women with endometriosis and/or adenomyosis were included in the above studies (intermittent oestrogen deprivation therapy, n = 77; low-dose or ‘draw-back’ therapy, n = 528; extended-interval dosing regimen, n = 171). However, almost half of these women (n = 335) participated in a single study comparing relugolix with leuprorelin at different doses (Harada *et al.*, 2022).

**Figure 1.**
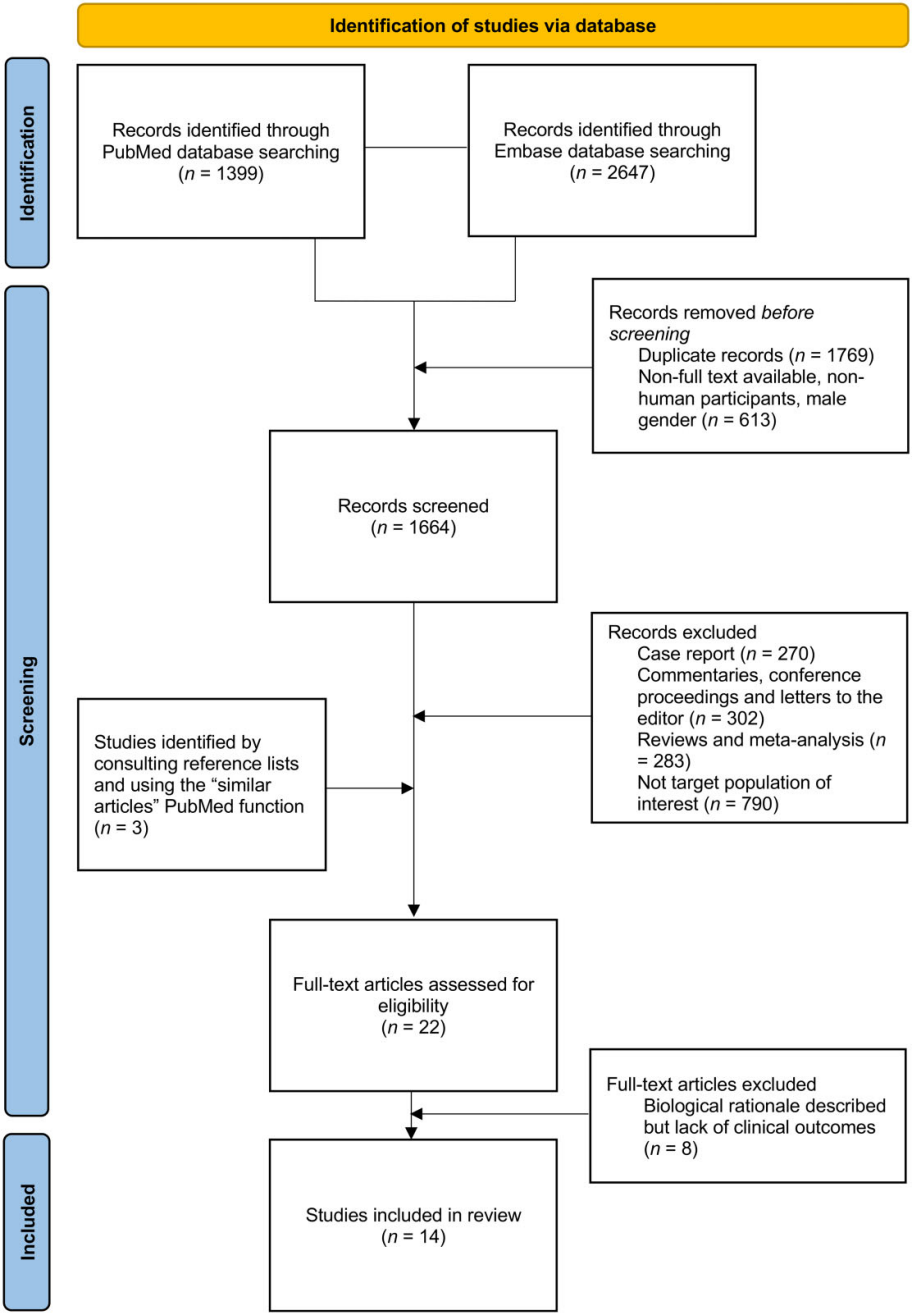
PRISMA flow diagram of the literature search and selection process.

Three potentially relevant articles were excluded as they were published in Chinese (Liu *et al.*, 2006, 2013) or Japanese (Tanaka and Umesaki, 2001) language journals. One of these studies focused on intermittent GnRH agonist treatment (Tanaka and Umesaki, 2001), one focused on low-dose, draw-back GnRH agonist therapy (Liu *et al.*, 2013), and one focused on an extended-interval GnRH agonist dosing regimen (Liu *et al.*, 2006). A total of eight articles addressing the biological rationale underpinning each individual alternative GnRH agonist use were identified but were excluded from the systematic review as clinical outcomes were not measured (Barbieri, 1992; Broekmans *et al.*, 1992; Henzl, 1992; Filicori *et al.*, 1998; Cheung *et al.*, 2000; Matteo *et al.*, 2006; Li *et al.*, 2014; Pohl *et al.*, 2022). The article selection process is described in Fig. 1.

### Intermittent oestrogen deprivation therapy

#### Biological rationale

The concept of intermittent oestrogen deprivation therapy for recurrent symptoms associated with endometriosis relapse dates back 30 years. In 1992, Henzl (1992) informed that a trial on re-treatment with a short nafarelin course was underway and reported some preliminary data. The idea was to administer a classic first-line 6-month course of a GnRH agonist (in this case, intranasal nafarelin, 200 µg/day b.i.d.) and then follow the patients, starting a second 3-monthly course of the same regimen only in cases of recurrence of moderate to severe pain symptoms.

According to this theory, a short re-treatment course could achieve the same clinical impact of the original 6-month course, while minimizing subjective hypo-oestrogenic effects and trabecular bone resorption. Indeed, Adamson *et al.* (1997) later maintained that ‘short (3-month) and frequent (as often as yearly if required) repeat applications of an intranasal GnRH agonistic analogue may be preferable to longer courses of therapy and greater suppression of estradiol levels, as well as to the need for estrogen “add-back” regimens’.

Although controversial, the therapeutic model of intermittent gonadal hormone deprivation is well-known in urological oncology for men with hormone-sensitive, locally advanced, relapsing or metastatic prostate cancer. The objectives of intermittent androgen deprivation are to improve quality of life by limiting the adverse events associated with sustained hormone suppression, and to reduce the cost of GnRH therapy. In these patients, intermittent, rather than continuous, GnRH agonist treatment aimed at decreasing testosterone production to castrate levels, may be guided by serum testosterone and prostate-specific antigen concentrations. According to the results of RCTs and systematic reviews, time to progression, cancer-specific survival, and overall survival appear to be similar in men allocated to intermittent or continuous androgen deprivation (Botrel *et al.*, 2014; Schulman *et al.*, 2016; Perera *et al.*, 2020).

Hypothetically, GnRH agonist re-treatment based on symptom recurrence makes empirical sense, as it is focused on and guided by the woman’s main clinical issue, i.e. pain. Furthermore, such an approach, by enabling cyclic recovery of oestrogen synthesis for variable time periods, could allow customization of therapy, preserve efficacy, improve safety, tolerability and quality of life, prevent over-medicalization, and reduce costs.

#### Clinical evidence

Hornstein *et al.* (1997) reported the effect of retreatment with intranasal nafarelin, 400 µg/day for 3 months, in 32 women who had already been treated for 6 months with the same regimen, and who experienced pain recurrence after a mean period of 10 months (range, 1–20 months). Pain scores decreased significantly by the end of the retreatment period and remained below baseline values 3 months after GnRH agonist discontinuation, despite a further trend towards worsening of symptoms over time. Lumbar BMD decreased by only 0.6 ± 0.5% after this 3-monthly nafarelin retreatment. A previous reduction of 1.4 ± 0.4% had been observed after the first 6-month nafarelin course.

The same year, Adamson *et al.* (1997) published the results of a repeated 3-month course of intranasal nafarelin, 400 µg/day, in 45 women with pain symptoms recurring after a first-line treatment with the same daily dose but used for 6 months. The second course was begun after >1 to <2 years from the previous treatment in 29% of the participants, and from 3 to <4 years in another 38%. Moderate to severe symptoms were reported in 89% of participants before retreatment. At the end of the repeated 3-monthly course, symptoms were absent or mild in 74% of patients and moderate in 26%. Three months after drug discontinuation, pain scores remained significantly lower compared with pre-retreatment values. Mean lumbar BMD decline was 1.9–2.2% at the end of the re-treatment period and 0.2–0.8% at 3-month follow-up assessment after drug discontinuation.

### Low-dose or ‘draw-back’ therapy

#### Biological rationale

Serum oestradiol (E2) concentrations below 20 pg/ml induce regression of endometriotic lesions and are generally associated with substantial pain relief, whereas serum E2 concentrations >60 pg/ml purportedly stimulate the growth of eutopic and ectopic endometrium (Barbieri, 1992). Based on the hypothesis that different organ tissues show varying sensitivity cut-offs to oestrogens (e.g. trabecular bone tissue is more sensitive than endometrial tissue), Barbieri suggested that the pharmacologic achievement of partial oestrogen suppression within a serum E2 range between 20 and 50 pg/ml, the so-called ‘therapeutic window’, would prevent endometriosis activation at the same time as minimizing vasomotor symptoms, genital atrophy, and a decrease in trabecular bone mineral density. The ‘oestrogen threshold hypothesis’ has guided much research on hormonal management of endometriosis even after three decades since its formulation (Pohl *et al.*, 2022).

However, one key assumption underpinning the ‘oestrogen threshold hypothesis’ or the ‘therapeutic window’ is homogeneity, but it has been consistently demonstrated that endometriotic lesions also produce oestrogens (see, as a review, Taylor *et al.*, 2021). Thus, the vast heterogeneity among endometriosis patients, due to different ages, reproductive history, co-morbidity, symptomatology, and disease severity, poses a great challenge to the ‘therapeutic window’ theory.

Nevertheless, several independent investigators developed what would later be called ‘draw-back therapy’, that is, initial achievement of complete pituitary desensitization using a standard GnRH agonist dose for a brief treatment period, and subsequent long-term tapering of the drug dose with the objective of maintaining a sufficient desensitizing effect that could prevent ovulation resumption but that, at the same time, could allow a basal gonadal function that would result in E2 production within the therapeutic window.

Other researchers directly assessed the effects of a lower than standard GnRH agonist dose without resorting to a starting full dose. Both approaches were expected to improve GnRH agonist safety, tolerability and adherence, sustain the antalgic effect over time and reduce costs, without resorting to add-back therapies.

#### Clinical evidence

In 1994, Hull and Barbieri (1994) were the first to alter a GnRH agonist dosage guided by serum E2 concentrations to limit pain symptom recurrence while mitigating oestrogen deprivation effects. They treated 23 women with endometriosis with intranasal nafarelin starting at the usual dosage of 400 µg/day (200 µg b.i.d.). After 4 weeks, the dosage was increased to 600 µg/day when the E2 level was >60 pg/ml and pelvic pain persisted, and it was reduced to 200 µg/day (or 200 µg every other day) if the E2 level was <20 pg/ml and oestrogen deprivation symptoms were reported. In seven women, the nafarelin dosage was reduced without pain recurrence or breakthrough bleeding episodes. However, pain intensity and BMD variations were not formally quantified before and after the 6-month study period.

The same year, Jacobson *et al.* (1994) reported the results of treatment of 25 patients with intranasal nafarelin at the dose of 200 µg/day (100 µg twice daily) for 6 months. In five participants, the dose had to be increased to the standard 400 µg/day regimen because of persisting menstruations. In the remaining patients, serum E2 levels were <60 pg/ml, amenorrhoea was maintained, and pain was significantly relieved despite incomplete pituitary desensitization induced by the halved nafarelin dose. In this case, treatment began upfront with a low GnRH agonist dose without an initial full-dose phase.

To verify whether nafarelin at low dose could be as effective as at full dose, and to assess the impact of combining the GnRH agonist with a low-dose progestin, Bergqvist *et al.* (1997) conducted a double-blind RCT on 47 women with laparoscopically confirmed endometriosis. Participants were allocated in a 1:2:1 ratio to nafarelin 200 µg/day plus oral placebo (n = 12), nafarelin 200 µg/day plus oral norethisterone acetate (NETA) 1.2 mg/day (n = 23), or nafarelin 400 µg/day plus oral placebo (n = 12) for 6 months. Nafarelin spray was used intranasally twice a day in all study groups, though at halved concentration in the two low-dose groups. Despite similar variations in serum E2 levels, pain symptoms decreased significantly only in the low-dose nafarelin plus NETA group and in the full-dose nafarelin plus placebo group. A marginally lower incidence of hot flushes and better bleeding control was observed in the combination therapy group. Pain symptom scores decreased similarly in the three study groups but, at the 6-month follow-up evaluation after drug discontinuation, remained lower than at baseline only in the combined therapy and full-dose nafarelin groups. Measurements of BMD variations during the study period were not performed.

To minimize the loss of bone mineral content without reducing the effect on pain symptoms, Uemura *et al.* (1999) started buserelin treatment in 21 women with endometriosis at the usual dose of 900 µg/day (1 spray in each nostril thrice daily) for 8 weeks, and then tapered the dosage to 600 µg/day (4 nasal sprays) for 16 weeks. During the study period, E2 levels remained below 60 pg/ml in 90% of the participants, pain was relieved in three out of four of them, breakthrough bleeding was almost avoided, but lumbar BMD decreased by 2.4 ± 0.5% at the end of treatment and by 1.1 ± 0.6% at 24 weeks after drug discontinuation.

Tahara *et al.* (2000) conducted a small, randomized pilot trial allocating 15 women with endometriosis to intranasal nafarelin at the standard 400 µg/day dose (i.e. 200 µg b.i.d.) for 24 weeks (n = 7) or to a standard dose for 4 weeks followed by half-dose (200 µg/day) for another 20 weeks (n = 8). In the experimental arm, serum E2 levels remained at ∼30 pg/ml despite the use of half nafarelin dose. No significant between-group differences in pain relief were observed, and only 25% of participants using low-dose nafarelin reported vasomotor symptoms compared with 86% of those using the full drug dose. After 24 weeks of treatment, lumbar mineral bone content loss was 1.4% in the half-dose group and 5.6% in the full-dose group.

Akira *et al.* (2009) treated 12 women with symptomatic adenomyosis for 2 years with a variable, reduced dose of intranasal buserelin acetate after a starting phase at full dose (900 µg/day, i.e. 6 nasal sprays/day). The dose of GnRH agonist was tapered to 150–750 µg/day, i.e. 1–5 sprays/day, based on maintenance of serum oestradiol levels (E2) between 20 and 50 pg/ml. The mean number of daily nasal sprays during the draw-back period was 2.9, corresponding to a mean daily buserelin dose of 435 µg. The mean serum E2 level was 36.3 ± 14.3 pg/ml. Chronic pelvic pain measurements dropped significantly compared with pre-treatment values and vasomotor symptoms were substantially mitigated during the draw-back phase compared with the initial full-dose buserelin phase. At the 6-month evaluation, the decrease in lumbar bone mineral density was 0.9 ± 0.9%.

Eight years later, Tang *et al.* (2017) reported the results of an RCT on the effect of a reduced depot GnRH agonist regimen. Leuprorelin was administered intramuscularly every 28 days for 24 weeks. A standard dose (3.75 mg) was used for the first and second injections and a half-dose (1.88 mg) was used for the remaining four injections in the 25 women in the experimental arm, whereas the standard dose only was used for all six injections in the 25 women in the control arm. The mean serum E2 concentration at 20-week assessment was 40.2 ± 8.7 pg/ml in the half-dose group and <20 pg/ml in the standard dose group. At the same time point, the mean lumbar BMD loss was 1.2% and 5.6%, respectively. Untoward hypo-oestrogenic effects were experienced significantly less frequently in women in the former than in the latter arm.

More recently, Harada *et al.* (2022) conducted a phase III, multicentre, double-blind, double-dummy RCT to assess the efficacy, safety and tolerability of oral relugolix, 40 mg/day (n = 171), compared with i.m. leuprorelin administered every 28 days (n = 164) at the dose of 3.75 mg in women weighing ≥50 kg (n = 116) and 1.88 mg in those weighing <50 kg (n = 48). No significant between-group differences were observed in the degree of pain relief, incidence of untoward effects and tolerability.

### Extended-interval dosing regimen

#### Biological rationale

The possibility of extending the interval of GnRH agonist administration pertains to sustained-release depot formulations only and is based on the hypothesis that commercially available ‘monthly’ preparations could maintain ovarian suppression for longer than the classic 28-day period for which they are licenced. The objective of extended-interval dosing regimens is reduction of cost and patient inconvenience.

Broekmans *et al.* (1992) assessed the pituitary and ovarian function after a single i.m. 3.75 mg depot triptorelin dose. The production of E2 gradually resumed during the 7th to 8th week after the injection and menstruations reappeared between the 11th and the 13th week. Filicori *et al.* (1998) confirmed that suppression of the pituitary–ovarian axis persists for about 2 months after injection of 3.75 mg triptorelin. These findings corroborate the notion that the use of depot triptorelin formulations may allow longer than currently indicated administration intervals. More recently, Li *et al.* (2014) confirmed that i.m. administration of depot triptorelin tends to suppress the pituitary–ovarian axis to a deeper degree compared to leuprorelin used with the same regimen.

Cheung *et al.* (2000) conducted a randomized, double-blind, crossover trial allocating participants with endometriosis to three doses of i.m. triptorelin 3.75 mg at 4-week intervals followed by three doses of i.m. leuprorelin 3.75 mg at the same time intervals (n = 27), or to the inverse sequence of the same two GnRH depot preparations (n = 21). The potency of both drugs in suppressing serum E2, FSH, and LH concentrations was similar, and the relative incidence and severity of untoward effects were comparable. However, the duration of pituitary down-regulation induced by triptorelin was substantially longer than that of leuprorelin. Time to spontaneous menstruation reappearance also was significantly longer after a last dose of triptorelin (leuprorelin-triptorelin sequence; 129 ± 7 days) than after a last dose of leuprorelin (triptorelin-leuprorelin sequence; 104 ± 5 days). This difference of over 3 weeks would allow depot triptorelin, but not depot leuprorelin, administration at substantially longer intervals than currently indicated. Pain symptoms and BMD variations were not measured.

With the objective of evaluating the duration of pituitary desensitization after a single i.m. injection of 3.75 mg of triptorelin or leuprorelin, Matteo *et al.* (2006) recruited 60 women with stages I–II endometriosis and administered a depot preparation of each GnRH agonist on the 21st day of the cycle to 30 participants. Based on serial weekly determinations of serum FSH, LH, and E2 levels, the two drugs similarly suppressed the pituitary–ovarian axis until the fourth week post-injection. From the fifth to the eighth week, both FSH and LH serum concentrations were higher in the leuprorelin group than in the triptorelin group. Complete inhibition of ovarian function was maintained until the sixth week after a single leuprorelin injection and until the seventh week after a single triptorelin injection. These findings confirm that the duration of down-regulation induced by a monthly depot injection of triptorelin is longer than that induced by the same dose of leuprorelin, and much longer than the 4-week interval currently adopted when treating patients with endometriosis.

#### Clinical evidence

Tse *et al.* (2000) reported, for the first time, the results of a small comparative study conducted on women with surgically diagnosed endometriosis undergoing a 6-month adjuvant triptorelin treatment. Depot 3.75 mg injections were administered every 4 weeks in the first five participants and every 6 weeks afterwards in 21 women. No between-group difference in hormonal suppression and pain symptoms improvement was observed. Serum E2 levels remained below postmenopausal levels (<150 pmol/l) up to 10 weeks after the last triptorelin injection, and menstruation resumed after a mean interval of 119–121 days.

Wong and Tang (2004) conducted an RCT on the effect of postoperative oral danazol, 600 mg/day (n = 20), versus triptorelin, 3.75 mg depot injections every 6 weeks (n = 20), for 6 months after surgery for stage III or IV endometriosis. No between-group difference in pain relief was observed, but triptorelin was better tolerated and was associated with a higher amenorrhoea rate than danazol. Serum FSH, LH, and E2 levels were consistently suppressed by triptorelin despite the extended interval between the four injected doses.

Kang *et al.* (2010) randomized 70 women with symptomatic adenomyosis or endometriosis to a 6-month treatment with 3.75 mg triptorelin injected i.m. every 6 weeks (n = 35) or every 4 weeks (n = 35). The authors did not observe significant between-group differences in the degree of pituitary–ovarian function suppression, induced amenorrhoea rate, pain score reduction, or frequency of side effects.

Liu *et al.* (2016) conducted a small parallel cohort study on 36 women who used postoperative triptorelin 3.75 mg for 24 weeks. No differences in E2, FSH, and LH serum levels were observed between eight women using the standard 4-week regimen, compared with 10 women using the standard dosage plus oral tibolone, 1.25 mg/day, and 16 women using the extended 6-week regimen. Time to resumption of menstruations was similar in the three study groups, being 81 ± 18 days in the conventional dosage regimen group and 76 ± 22 days in the extended-interval group. No differences were observed in pain symptom relief.

The above findings confirm that an extended-interval triptorelin dosing regimen ensures identical clinical effects compared with the standard-interval dosing regimen, while cutting costs by one-third and potentially increasing treatment acceptance and adherence.

## Discussion

The quality of the evidence on the effect of the three considered alternative clinical approaches based on a biological rationale and aimed at limiting the cost of treatment with GnRH agonists, appears suboptimal. Most studies were small, and some were also rather dated, mainly non-comparative and retrospective. Even when randomization was adopted for treatment allocation, the sample size was too limited to exclude the risk that potentially important between-group differences went unrecognized. Symptoms were not always reliably measured, or non-validated scales were used. Criteria for the definition of symptom recurrence sometimes were unclear, and the length of follow-up was generally short.

**Figure 2.**
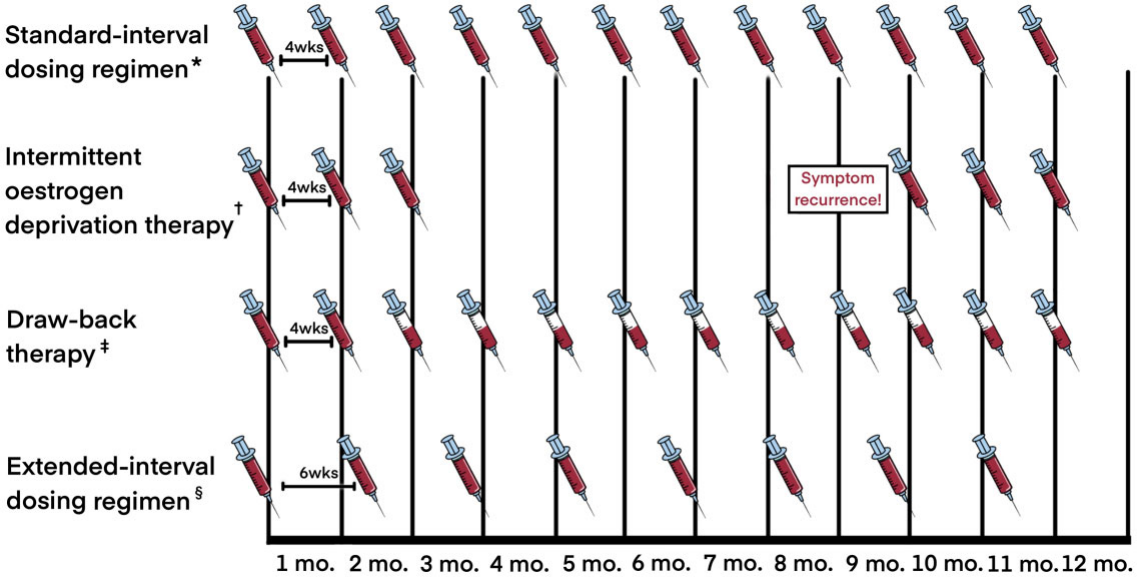
**Simplified schematic representation of the evaluated alternative modalities for the use of monthly depot GnRH agonist preparations considering a hypothetical 12-month treatment period.** The number of doses is approximate because depot GnRH agonist preparations should be injected every 28 days, not every month. Full syringe: standard-dose monthly depot GnRH agonist preparation. Half-full syringe: low-dose monthly depot GnRH agonist preparation. *Standard-interval dosing regimen using any monthly depot GnRH agonist preparation injected every 4 weeks. ^†^Based on a 3-month first-line course of standard-dose monthly depot GnRH agonist preparation followed by a 3-month second-line course using the same regimen due to hypothetical symptom recurrence.^‡^Two months of standard-dose monthly depot GnRH agonist followed by the same monthly regimen at half dose.^§^Extended-interval dosing regimen using only triptorelin 3.75 mg depot preparation injected every 6 weeks instead of every 4 weeks.

Nonetheless, some conclusions may still be drawn on the possibility of mitigating the expenditure for GnRH agonists when these second-line medications are indicated in women with progestogen-resistant endometriosis (Table I and Fig. 2).

**Table I.** Potential strategies to mitigate the financial burden of GnRH agonist treatment for progestogen-resistant endometriosis; literature data, 1992–2022.

Strategy type	Advantages	Disadvantages	Supporting studies	1-year treatment cost*	% cost reduction
Intermittent 3-monthly GnRH agonist courses (intermittent oestrogen deprivation therapy)	Limited duration of side effects with potential increase in acceptability and adherence; reduced bone resorption; treatment only as required, avoidance of needless prolonged ovarian suppression	Psychologically distressful, with focus on symptom recurrence; uncertain effect on deep dyspareunia; potential unrecognized deep lesion progression and endometrioma development/recurrence	Hornstein *et al*, 1997 Adamson *et al*., 1997	Variable, based on frequency of 3-monthly GnRH agonist courses	Variable, owing to increased need for clinical and sonographic assessments; add-back therapy cost to be added when multiple courses needed
Low-dose, draw-back therapy	Reduced incidence and severity of side effects, including the magnitude of bone resorption	Uncertainty of ovulation inhibition, risk of unrecognized conceptions; need for manual preparation of half-dose if depot formulation used	Hull and Barbieri, 1994 Jacobson *et al*., 1994 Bergqvist *et al*., 1997 Uemura *et al*., 1999 Tahara *et al*., 2000 Akira *et al*., 2009 Tang *et al*., 2017 Harada *et al*, 2022	Unvaried if half-dose depot preparation not marketed; nasal sprays no longer popular and currently not always available	Not applicable
Extended-interval dosing regimens	Maintenance of a stable, low-oestrogenic environment, with predictable amelioration of pain and progressive improvement of dyspareunia	Prudential need for barrier contraception; choice of GnRH agonist limited to depot triptorelin because of specific prolonged duration of action	Tse *et al*., 2000 Wong and Tang, 2004 Kang *et al*., 2010 Liu *et al.*, 2016	€1485.55^†^ £1306.62^‡^ $1579.38^‡^ €114 to be added for standard add-back therapy^§^	33% compared standard 4-week interval regimen; 31% including the cost of add-back therapy

* Based on Italian market prize, February 2023.

† Based on a 6-week extend-interval dosing regimen and eight injections of 3.75 mg triptorelin per year.

‡ Based on exchange rates on 22 February 2023.

§ Cost of 365 days of treatment with a standard add-back therapy containing oestradiol 1 mg and norethisterone acetate 0.5 mg in Italy (€8.77 for a 28-day tablet package).

Regarding intermittent oestrogen deprivation therapy, retreatment for 3 months seems to achieve substantially similar effects compared with that for 6 months (Hornstein *et al.*, 1995). Consequently, repeated 3-monthly, GnRH agonist monotherapy courses could be planned at symptom reappearance only, instead of prolonging treatment indefinitely and combining GnRH agonists with add-back therapy (Adamson *et al.*, 1997).

However, as pain recurrence after GnRH agonists withdrawal should be expected in most women, the need for retreatment would be frequent. This would imply an overall high number of months under oestrogen deprivation. Therefore, GnRH agonists could not be used as monotherapy, and combination with oestrogen–progestogens would be needed anyway (Leone Roberti Maggiore *et al.*, 2014).

Moreover, prolonged ovulation resumption between treatment courses would increase the risk of endometrioma development or recurrence (Vercellini *et al.*, 2008, 2009b) and increase the risk of deep lesion progression (Netter *et al.*, 2019). This could be particularly hazardous when infiltrating endometriotic nodules are close to the pelvic ureter or determine bowel stenosis not yet causing sub-occlusion (Vercellini *et al.*, 2021). Thus, women should undergo frequent follow-up visits and ultrasound scans when not using GnRH agonists. This would increase healthcare resources utilization and the burden of treatment as well. Finally, focusing on symptoms in the attempt of identifying disease relapse as quickly as possible, may be psychologically distressful as it puts a woman’s complaints at the centre of her everyday life.

The draw-back therapy model was initially proposed when nasal sprays were a common modality to administer GnRH agonists. However, independently of the inconvenience and additional costs related to serial serum E2 and FSH determinations needed for dose modulation, these formulations are no longer available. Administering lower than standard doses of GnRH agonist is still suggested by some investigators when using depot formulations (Tang *et al.*, 2017). However, as low-dose GnRH agonist depot preparations are not marketed, this approach implies using only half of a standard package drug content. Clearly, this does not allow savings, and the patient is left with potential disadvantages only. In fact, subjective dosing may be imprecise and increase the risk of unpredicted ovulations. This could result in suboptimal disease control or unrecognized pregnancies, as it has been the case with the GnRH antagonist elagolix (Taylor *et al.*, 2017). Therefore, on one hand, the use of non-hormonal contraception is mandatory, thus potentially affecting treatment adherence, and on the other hand, add-back therapy cannot be avoided anyway, as bone mineral density declines even if ovarian steroidogenesis is not completely inhibited.

Using depot formulations according to the extended-interval dosing regimen seems the most feasible and realistic option among the three considered alternatives to contain the expenditure for GnRH agonist treatments. Pharmacokinetics aspects play a crucial role here, as the duration of action of depot leuprorelin and triptorelin preparations is different. In fact, using the standard 3.75 mg dose, only the latter medication allows consistent gonadal suppression for at least 6 weeks (Broekmans *et al.*, 1992; Filicori *et al.*, 1998; Cheung *et al.*, 2000; Tse *et al.*, 2000; Wong and Tang, 2004; Matteo *et al.*, 2006; Kang *et al.*, 2010; Li *et al.*, 2014; Liu *et al.*, 2016). Therefore, 3.75 mg depot triptorelin injections every 6 weeks instead of 4, would allow substantial savings without compromising efficacy, as irregular ovulations and erratic bleeding episodes would be prevented.

In Italy, one 3.75 mg depot triptorelin injection costs €171.10. This translates to a daily cost of €6.11 when injecting triptorelin every 28 days, and of €4.07 when injecting the drug every 42 days. Thus, 1-year treatment with the standard modality sums up to €2230.15, whereas with the extended-interval dosing regimen, the total cost is €1485.55 (−744.60; −33.4%).

For those women who prefer a schedule with even longer intervals between injections, triptorelin 11.25 mg depot preparations could be used. In fact, Donnez *et al.* (2004) reported the results of a multicentre, phase II RCT conducted on women with surgically confirmed endometriosis who received a single i.m. injection of 3-month slow-release triptorelin (n = 72) or a standard 3.75 mg triptorelin injection every 28 day for 3 months (n = 74). In women receiving the 3-month preparation, mean serum oestradiol levels remained suppressed for a significantly longer period compared to women receiving the standard 28-day injections. On Day 112, median oestradiol concentrations were 10.3 pg/ml in the 3-month group and 46.5 pg/ml in the 28-day group. Corresponding median values on Day 140 were 48.7 and 70.2 pg/ml, and the mean castration (E2 < 50 pg/ml) duration was 122 and 94 days, respectively. Accordingly, menses returned after a mean period of 5.1 months in the triptorelin 11.25 mg group and after 4.4 months in the standard 3.75 mg group.

Based on the above data, an extended-interval dosing regimen could be hypothesized also for the 11.25 mg depot triptorelin preparation, which could be safely administered every 4 months instead of 3 months. In Italy, this would limit the cost of treatment by 25%, reducing the yearly expenditure for this GnRH agonist from €1993.44 (498.36 × 4) to €1495.08 (498.36 × 3). This figure is almost identical to the €1485.55 needed for 365 days of treatment with triptorelin 3.75 mg injections administered every 6 instead of 4 weeks. Nevertheless, to our knowledge, clinical data on an extended-interval dosing regimen using the 11.25 mg depot formulation have not been published.

As add-back therapies cannot be avoided with extended-period dosing regimens, their cost must be added to that of triptorelin. Oral tibolone, 2.5 mg/day (in Italy, €125 per year), or a combination of oestradiol 1 mg plus NETA 0.5 mg (in Italy, €114 per year), have been demonstrated to be safe, effective, and well-tolerated (Berning *et al.*, 2001; Castrejón-Delgado *et al.*, 2021). These add-back therapies maintain pain relief, prevent breakthrough bleeding and minimize bone loss (Compston, 1995; Lindsay, 1996).

Relugolix, an oral GnRH antagonist, has been licenced by the European Medicine Agency for the treatment of fibroids, but it will soon be approved for endometriosis as well. Drug cost should be included in the overall balance that could be used to counsel women and should allow informed choices when the use of GnRH agonists or antagonists is indicated. In Italy, the cost of 1-year treatment with oral relugolix combined with add-back therapy is €2153.50 (28 tablets, €165.10 = €5.90 per day; £1894.11; $2289.53), whereas the yearly cost of treatment with triptorelin 3.75 mg injected every 6 weeks plus the same add-back therapy used with the commercially available relugolix combination therapy (oral oestradiol, 1 mg plus NETA 0.5 mg; €114; £100.27; $121.20) amounts to €1599.55 (£1406.88; $1700.59). Considering that no differences in efficacy, safety, and tolerability have been demonstrated between relugolix and GnRH agonists (Harada *et al.*, 2022), adopting the combined extended interval triptorelin dosing regimen would result in the same overall clinical impact as that observed with the combined relugolix treatment, but with a saving of €554 (£487.27; $588.99). This corresponds to a one-quarter reduction in yearly drug cost (−25.7%). Conversely, if triptorelin is injected every 28 days as usual, the yearly cost of the GnRH agonist (€2230.15) plus add-back therapy (€114) totals €2344.15 (£2061.8; $2492.22), with an extra-expenditure of €190.65 (£167.69; $202.69) compared with the combined relugolix therapy.

This systematic review has some limitations. The available evidence was not entirely considered, as reports not published in English language journals and conference proceedings were excluded. No attempt was made to identify unpublished studies. Moreover, the quality of the evidence reported in the selected articles was not formally evaluated. Furthermore, owing to the extreme qualitative heterogeneity in study characteristics, a quantitative synthesis was not performed. In addition, some relevant studies were old and so the tested therapeutic approach is apparently obsolete, or the GnRH agonist used is no longer on the market in many countries.

Another obvious limitation of this review is that only cost containment associated with GnRH analogue use has been analysed, and not cost-effectiveness, which is a much more complex but also more useful measure within the context of health technology assessment. To avoid the potential impact of financial conflicts of interest and ensure equity towards other patients at large, comparative effectiveness research and cost-effectiveness analyses in the field of pharmacological management of endometriosis should be conducted by independent investigators (Xie and Zhou, 2022). Meanwhile, a stepped-care approach should be adopted in women with endometriosis, and expensive GnRH analogues used exclusively when progestogens are found to be ineffective, not tolerated or contraindicated (Vercellini *et al.*, 2018a,b,c; Becker *et al.*, 2022).

Oral contraceptive pills used continuously are very frequently prescribed also as a measure to contain costs especially in low-resource settings. In this regard, we could have also included this treatment alternative in our review. However, our objective was to address the most studied second-line treatment to be used precisely when first-line medications such as low-dose oral contraceptives fail. In fact, non-response to progestogens is not uncommon. According to Taylor *et al.* (2021), progestogens and low-dose oral contraceptives are unsuccessful in about a third of symptomatic women, presumably owing to progesterone resistance. Treatment with GnRH agonists could allow adequate symptom relief and disease control in this subset of endometriosis patients, although further research is needed.

Mitigating the financial burden of GnRH agonist treatment must never be negotiated with suboptimal patient care. At the same time, trying to make the most of the scarce resources available is not and should not be intended as rationing, but as a determinant of a well-balanced, inclusive and equitable management of publicly funded healthcare systems (Whitehead, 1992). When a patient with endometriosis needs a second-line medication, the GnRH analogue (agonist or antagonist) that combines optimal efficacy with the lowest possible expenditure should be chosen. Based on a large amount of published data, today this appears possible adopting the extended-interval dosing regimen with the use of 3.75 mg depot triptorelin formulations plus add-back therapy. This would allow saving of about one-third of the overall yearly cost of such medical treatment. Why not?

## Data Availability

Data included in this article were extracted as published in available original articles. No new data were generated or analysed in support of this review.
